# Rheological properties of warm mixed high viscosity asphalt at high and low temperatures

**DOI:** 10.1371/journal.pone.0301138

**Published:** 2024-03-28

**Authors:** Jun’an Lei, Nanxiang Zheng, Yuanyuan Wang, Haicheng Su, Xiaofeng Ren, Fujing Zhao

**Affiliations:** 1 School of Civil Engineering and Architecture, HuBei University of Arts and Science, Xiangyang, Hubei, China; 2 School of Highway, Chang’an University, Xi’an, Shaanxi, China; 3 Xiangyang Road and Bridge Construction Group Co., Ltd., Xiangyang, Hubei, China; Beijing University of Technology, CHINA

## Abstract

The rheological properties of asphalt can well reflect its road performance, but the rheological properties of warm mix high viscosity asphalt (HVA) are unclear. In order to study the effect of warm mixing agent on rheological properties of HVA, two kinds of warm mixing agent EC120 (EC) and Evotherm M1 (M1) were selected to prepare warm mix HVA. The rheological properties of warm mix HVA at high temperature (135~195°C), medium temperature (0~80°C) and low temperature (-6~18°C) were studied by Brinell rotary viscosity test, dynamic shear rheological test (including temperature scanning, frequency scanning, linear amplitude scanning) and bending beam rheological test. The test results show that both EC and M1 have good viscosity reduction effect on HVA at high temperature, and can effectively reduce the construction temperature. At medium temperature, M1 can effectively improve the fatigue resistance of HVA, and the fatigue life can be increased by about 30% when the dosage is 0.6%. EC can increase the rutting factor of HVA and improve its resistance to deformation, but it will reduce its fatigue performance. When the dosage is 4%, the fatigue life will be reduced by about 9%. At low temperature, M1 can reduce the creep stiffness S, increase the creep rate m, and improve the low temperature performance of HVA, while EC has the opposite effect, weakening the low temperature performance of HVA. The results are helpful to understand the rheological properties of warm mix HVA and promote its application.

## 1 Introduction

Porous asphalt pavement has the advantages of high water permeability, good skid resistance in rainy days and low tire noise, which can significantly improve driving safety and comfort [1, 2]. Due to the large void ratio of the porous asphalt pavement, the asphalt will age under natural conditions such as light, rain and temperature, which will easily lead to the diseases such as loose and peeling of the pavement. The high viscosity modified asphalt has the advantage of strong cohesion [3–5], which is suitable for use in large void asphalt mixture, and can effectively prolong the service life of drainage pavement. However, due to its high viscosity, the mixing temperature of asphalt will be greatly increased, which is easy to cause problems such as asphalt aging [6] and environmental pollution [7]. Warm mixing technology [8, 9] can effectively reduce the temperature of asphalt during construction. Combining warm mixing technology with high viscosity asphalt can reduce the aging of asphalt and reduce the emission of harmful gases.

According to the mechanism of asphalt warm mixing technology, it can be divided into three categories [10–12], namely foam viscosity reduction technology [13], organic additive technology [14] and surface active warm mixing technology [15]. The principle of foam warm mixing technology is that foam asphalt is obtained by adding cold water to hot asphalt by foaming machine, so as to improve the construction workability of asphalt mixture. Organic warm mixing technology is to add organic warm mixing materials into asphalt to achieve the purpose of reducing the viscosity of asphalt. Surface active warm mixing technology is to reduce the mixing temperature of asphalt mixture by adding surfactants. Gao ZW [16] and Zhang JT [17] studied the effect of warm mixing agent on SBS modified asphalt and found that the viscosity of asphalt was reduced and the high-temperature performance was improved. Akisetty [18, 19], Wang HN [20] and Turbay E [21] studied the effect of warm mixing agent on the properties of rubber asphalt and found that the permanent deformation of asphalt was reduced. Li ZX [22] and Yu X [23] studied the rheological properties of the warm-mixed recycled asphalt, and the results showed that the warm-mixed recycled asphalt exhibited better high-temperature performance. Behl A [24] studied the aging properties of warm-mixed asphalt and found that warm-mixed asphalt had stronger anti-aging properties. Liu QT [25] found that the fatigue resistance of warm-mixed asphalt was slightly higher than that of hot-mixed asphalt mixture. The existing research shows that the warm mixing agent can improve the high and low temperature properties and the anti-aging properties of asphalt.

Although there are many researches on the properties of warm mix asphalt, they mainly focus on matrix asphalt, SBS modified asphalt and recycled asphalt. There is a lack of research on the effect of warm mix agents on the performance of high viscosity asphalt. In addition, the rheological properties of asphalt can well reflect its road performance [26]. The existing research only focuses on the rheological properties of asphalt at specific temperatures, without systematically comparing the rheological properties of HVA under high, medium, and low temperature conditions.

In this study, two kinds of warm mixing agent, organic additives and surfactants, were selected to prepare warm mix HVA. By using Brinell rotary viscosity test, dynamic shear rheological test and bending beam rheological test, the effects of the type and amount of warm mixing agent on the rheological properties of HVA at high, medium and low temperatures were studied, which provided a useful reference for improving the application of high viscosity asphalt.

## 2 Materials

### 2.1 High viscosity asphalt

The high viscosity asphalt used in this study is prepared by adding 8% TPS high viscosity agent to SBS modified asphalt, and its basic technical indicators are shown in Table 1.

**Table 1:** Basic properties of HVA.

Item		Unit	Requirement	Result	Test method
Penetration (25°C, 100g, 5s)		0.1mm	≥40	47.70	T0604
Softening point		°C	≥80	100.45	T0606
Flash point		°C	≥260	296	T0611
Solubility		%	≥99	99.3	T0607
Brinell viscosity (135°C)		Pa•s	--	4.125	T0625
Dynamic viscosity (60°C)		Pa•s	≥50000	896100	T0620
Elastic recovery (25°C)		%	≥95	99.78	T0662
After RTFOT	Mass change rate	%	±0.2	0.13	T0609
	Penetration residual rate	%	≥65	84.14	T0604

### 2.2 Warm mixing agent

In this study, two different types of warm mixing agents were selected for research, namely EC120 (EC) and Evotherm M1 (M1), as shown in Table 2.

**Table 2:** Types of warm mixing agents.

Type	Producer	Physical state	Colour	Dosage	Working mechanism
EC	China	Solid powder	White	2%	Organic warm mixing technology
				4%	
				6%	
M1	America	Liquid	Dark brown	0.3%	Surface active warm mixing technology
				0.6%	
				0.9%	

EC is a product of Haichuan Company in Shenzhen, China. It is a synthetic straight chain aliphatic hydrocarbon with a white granular appearance. It is insoluble in water and has a melting point of around 100°C. When the temperature is higher than 110C, it will completely dissolve in asphalt, presenting a grid structure. This can help EC stabilize well in asphalt without segregation.

M1 is the third generation product of MeadWestvaco (MWV), a dark yellow brown viscous liquid that can be directly added to asphalt to reduce the construction temperature of the mixture. Its working principle is to change the polarity between asphalt molecules through surfactants, change the surface tension between asphalt molecules and aggregates to achieve the purpose of warm mixing. Compared with the hot mix asphalt mixture, the construction temperature can be reduced by 20°C~50°C.

### 2.3 Preparation of warm mix high viscous asphalt

**Fig 1:**
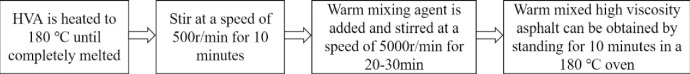
Preparation process of warm mixing high viscosity asphalt.

The preparation process of warm mix high viscosity asphalt is shown in Fig 1. Firstly, the HVA is heated to about 180°C, and after completely melting, the shear machine is used to stir at a speed of 500r/mim for 10min. Then the warm mixing agent is added to the asphalt and stirred at a speed of 5000r/min for 20–30 minutes. Finally, the warm mixed high-viscosity asphalt can be obtained by standing in the oven at 180°C for 10min.

## 3 Experimental

**Fig 2:**
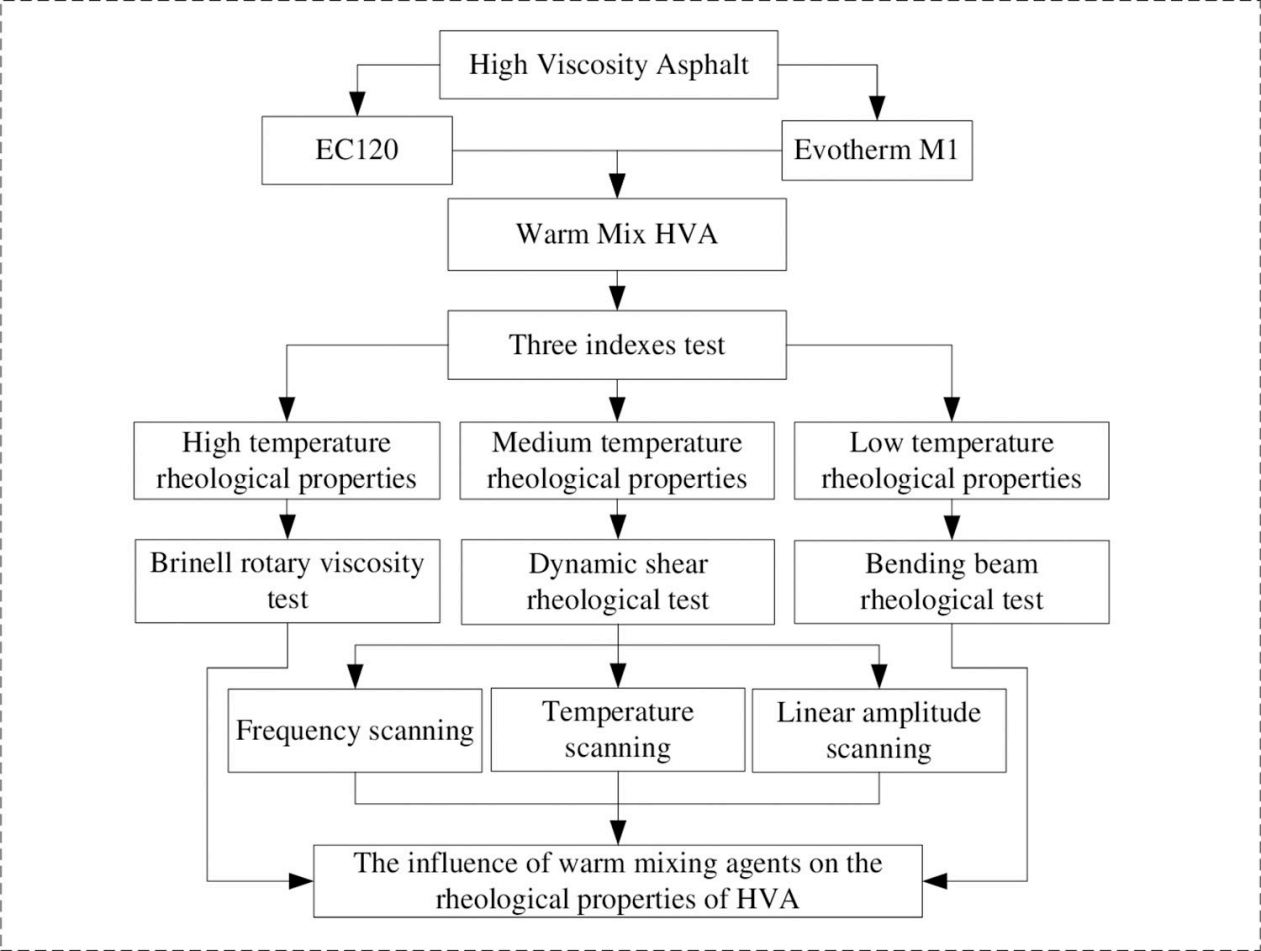
Flow chart scheme of this paper.

The experimental route of this study is shown in Fig 2. Firstly, the warm mix HVA asphalt was prepared. Secondly, the rheological properties tests of asphalt under high, medium and low temperatures were conducted, including Brinell rotational viscosity test, dynamic shear rheological test, and bending beam rheological test. Finally, the influence of warm mixing agents on the rheological properties of HVA was analyzed.

### 3.1 Three indexes test

The three indexes of asphalt include penetration, softening point and ductility, which are the most basic indexes to evaluate the high and low temperature properties of asphalt binder. The penetration and softening point reflect the high temperature performance of asphalt, and the ductility reflects the low temperature performance of asphalt. The test method was carried out according to JTG E20-2011.

### 3.2 Rotational viscosity test

**Fig 3:**
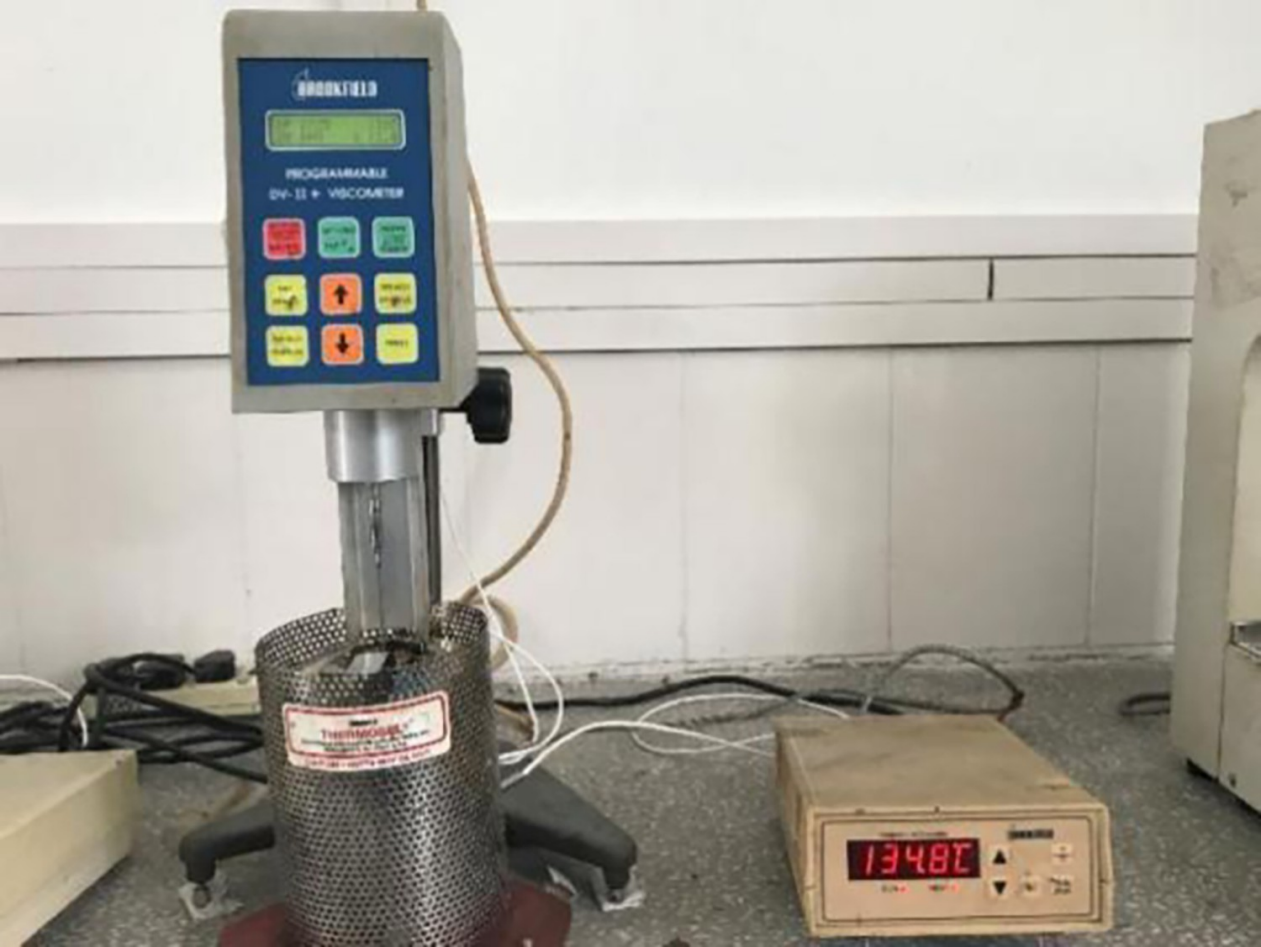
Brockfield viscosity test.

The Brookfield rotary viscosity tester was used to test the viscosity of HVA and warm mix HVA respectively at 135°C, 150°C, 165°C, 180°C and 195°C, As shown in Fig 3. The test method was carried out according to JTG E20-2011.

### 3.3 Dynamic shear rheological test

**Fig 4:**
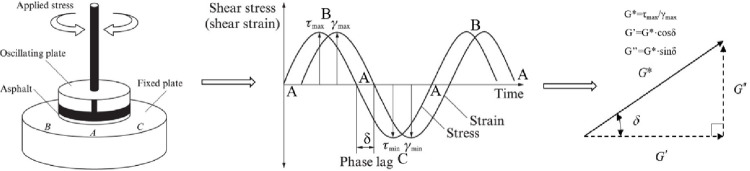
The working principle of DSR.

The rheological properties of asphalt were studied by dynamic shear rheometer (DSR), as shown in Fig 4. The oscillating plate rotates along A-B-A-C-A to form one cycle. The variation curve of stress or strain with loading time can be obtained. Then the rheological properties of asphalt such as complex shear modulus G*, phase angle δ, rutting factor G*/sinδ, fatigue factor G*·sinδ were calculated.

The complex shear modulus G* is the ratio of the peak shear stress *τ*_max_ to the peak shear strain *γ*_max_, which represents the resistance to deformation of the material under repeated stress. The larger the value, the stronger the resistance to deformation. G* is composed of two parts: storage modulus G’, which represents elastic properties, and loss modulus G”, which represents viscous properties. The storage modulus G’ reflects the energy storage and release in the process of asphalt deformation, and the larger the value, the stronger the energy storage capacity and the recovery capacity of asphalt. The loss modulus G” reflects the energy lost in the form of heat due to internal friction in the process of asphalt deformation, which cannot be recovered. The phase Angle δ represents the time lag between shear stress and shear strain. The smaller the phase Angle is, the greater the proportion of elastic part of the material, and the deformation is easier to recover after the applied load is withdrawn. G*/sinδ is the rutting factor, which is used to represent the permanent deformation resistance of asphalt material. The larger the value, the smaller the flow deformation of asphalt, and the less rutting is generated on asphalt pavement. G*·sinδ is the fatigue factor, and the smaller the value, the better the anti-fatigue performance of asphalt.

Three modes of temperature scanning, frequency scanning and linear amplitude scanning can be set during rheological performance testing.

#### 3.3.1 Temperature scanning

Temperature scanning is used to study the rheological properties of asphalt by changing the test temperature. When the temperature scanning range is -9°C~30°C, the rotor diameter is 8mm, the rotor clearance is 2mm, the shear strain is 0.1% (strain control mode), the shear rate is 6.283rad/s, and the heating rate is 2°C/min. When the temperature scanning range is 30°C~80°C, the rotor diameter is 25mm, the rotor clearance is 1mm, the shear stress is 100Pa (stress control mode), the shear rate is 10rad/s, and the heating rate is 2°C/min.

#### 3.3.2 Frequency scanning

Frequency scanning is used to study the rheological properties of asphalt by changing the test frequency. In reality, the road condition will be affected by the driving speed, the faster the driving speed, the greater the loading frequency. In order to simulate the influence of frequency on asphalt, DSR was used to perform frequency scanning test on asphalt at 60°C. The scanning frequency range is 0.01rad/s~100rad/s, the control strain is 1%, the rotor diameter is 25mm, and the rotor clearance is 1mm.

#### 3.3.3 Linear amplitude scanning

Fatigue damage of asphalt will accumulate gradually with loading time. The anti-fatigue properties of asphalt can be studied by linear amplitude scanning (LAS). The LAS experiment was conducted based on DSR. LAS test can realize the accelerated development and accumulation of asphalt damage by linearly increasing the applied shear strain amplitude under constant test temperature and loading frequency, thus improving the efficiency of asphalt fatigue property measurement. The asphalt used in LAS test is the residue after rotary film oven aging (RTFOT) and pressure aging (PAV). The rotor plate of LAS test is 8mm in diameter, 2mm in spacing, and 15°C in temperature during the test.

**Fig 5:**
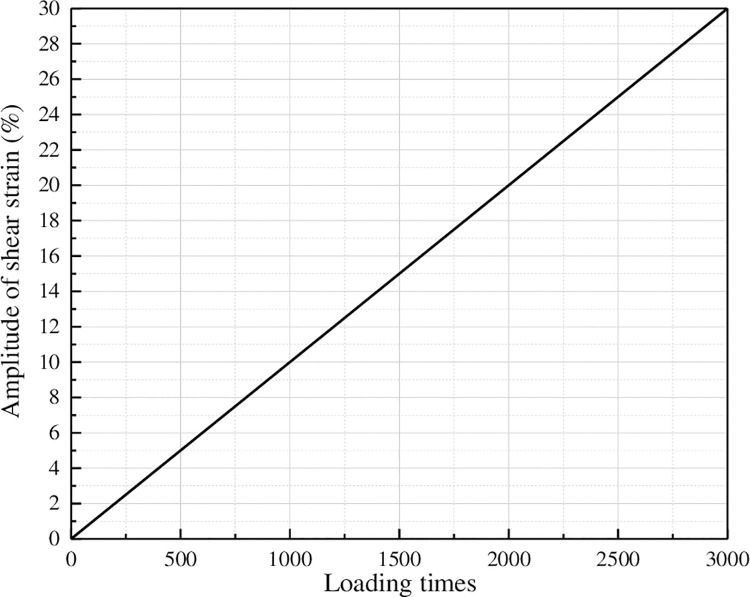
Loading scheme of amplitude scanning test.

The LAS test is divided into two steps, namely frequency scanning and amplitude scanning. In the first step, DSR is used to perform frequency scanning test under controlled strain mode. The strain is constant at 0.1%, and the scanning frequency ranges from 0.2Hz to 30Hz, referring to the following 12 specific frequencies: 0.2, 0.4, 0.6, 0.8, 1.0, 2.0, 4.0, 6.0, 8.0, 10, 20 and 30Hz. The dynamic shear modulus G* and phase Angle δ of asphalt at each frequency were recorded during the test. The second step is amplitude scanning test, which is carried out in strain control mode. The loading frequency is 10Hz, and the strain is loaded for 10 seconds (that is, 100 cycles) under different strain amplitudes. The strain increases linearly from 0.1% to 30%, as shown in Fig 5. During the test, the shear stress peak, shear strain peak, dynamic shear modulus G* and phase Angle δ were recorded every 10 loading cycles (1s).

The storage modulus G’ is calculated based on dynamic shear modulus and phase Angle at different loading frequencies.


$$G^{\prime }(\omega )=|G^{*}|(\omega )•\cos\delta (\omega )$$ (1)

With log(ω) as the horizontal coordinate and logG’(ω) as the vertical coordinate, the Formula (2) can be obtained by linear fitting of the test data.


$$\log G^{\prime }(\omega )=m\log\omega +b$$ (2)

Based on the m value obtained by fitting, the lossless characteristic parameter α is calculated according to Formula (3).


$$\alpha =\frac{1}{m}+1$$ (3)

The approximate calculation method of cumulative damage (D) of asphalt samples is shown in Formula (4).


$$D(t)≅{\sum_{i=1}^{N}\left[\pi I_{D}\gamma_{0}^{2}(|G^{*}|\sin\delta_{i-1}-|G^{*}|\sin\delta_{i})\right]}^{\frac{\alpha }{\alpha +1}}{\left(t_{i}-t_{i-1}\right)}^{\frac{1}{\alpha +1}}$$ (4)

Where: I_D_ is the dynamic shear modulus measured by 1% strain, MPa; γ_0_ is the shear strain of a given calculated data point, %; G* is the measured dynamic shear modulus, MPa; α is the lossless characteristic parameter calculated by Formula (3); t is the test time, s.

The accumulation of damage starts from 1% strain amplitude data points up to 30% strain amplitude. For each test data point, the calculation results of |G*|•sin*δ* and *D*(*t*) were recorded separately. Assuming *D*(0) = 0, and the initial value of |G*|•sin*δ* is equal to the average measured value of |G*|•sin*δ* in the 0.1% strain amplitude interval, and then |G*|•sin*δ* and *D*(*t*) are fitted according to Formula (5).


$$\log(C_{0}-|G^{*}|•\sin\delta )=\log C_{1}+C_{2}\log D(t)$$ (5)

Where: C_0_ is the average value of |G*|•sin*δ* measured in the 0.1% strain amplitude interval; C_1_ is the fitting parameter, which can be calculated from the intercept log *C*_1_; C_2_ is the slope of the fitted equation. In the calculation of C_1_ and C_2_, the data of *D*(*t*) can be ignored when it is less than 100.

The cumulative damage value D_f_ of asphalt when fatigue failure occurs is defined as the *D*(*t*) value when |G*|•sin*δ* decays 35% from the initial value of the lossless state.


$$D_{f}=0.35{\left(\frac{C_{0}}{C_{1}}\right)}^{\frac{1}{C_{2}}}$$ (6)

The fatigue life of asphalt binder is calculated according to Formula (7).


$$N_{f}=A_{35}{\left(\gamma_{\max}\right)}^{-B}$$ (7)

Where, A_35_ and B are parameters in the asphalt fatigue model, which can be calculated according to Formulas (8) and (9).


$$A_{35}=\frac{f•{\left(D_{f}\right)}^{k}}{k{\left(\pi I_{D}C_{1}C_{2}\right)}^{\alpha }}$$ (8)

B=2α (9)

Where: f is the loading frequency, 10Hz; k = 1+(1-C2)α; γ_max_ is the maximum shear strain of asphalt in pavement, %.

### 3.4 Bending beam rheological test

**Fig 6:**
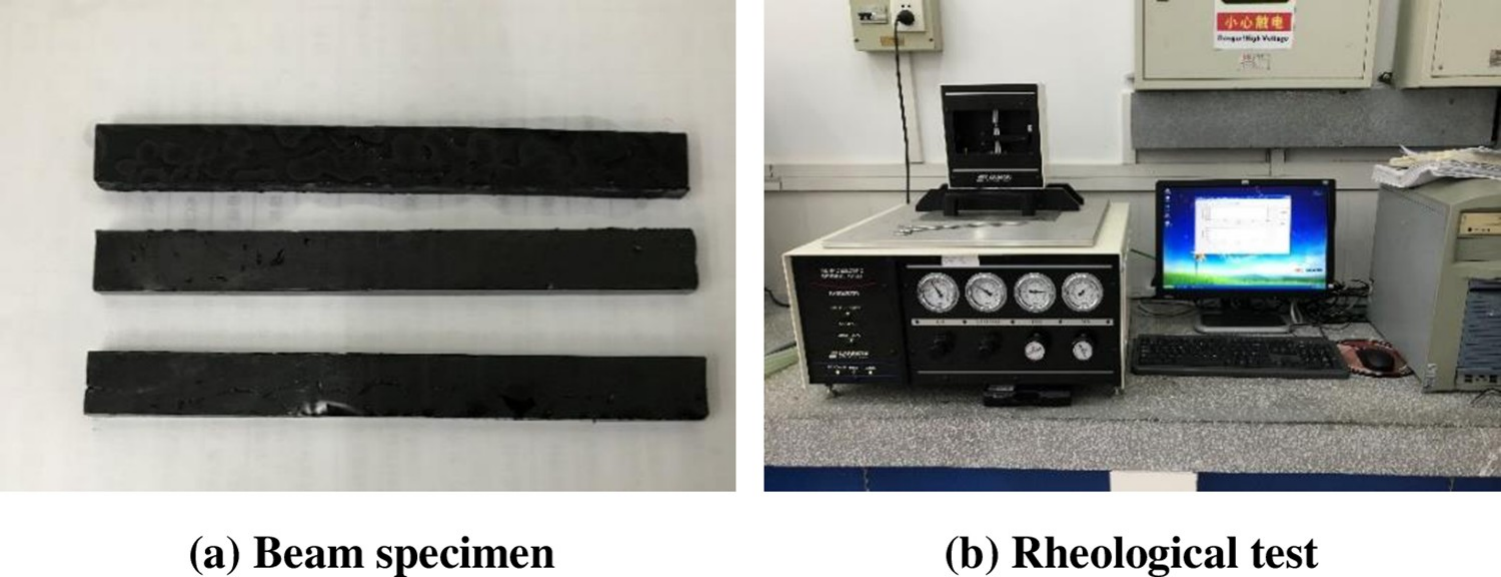
Bending beam rheological test.

The bending beam rheological (BBR) test was used to evaluate the low temperature properties of asphalt. Asphalt is the residue after RTFOT and PAV. The size of the specimen is 127mm×12.7mm×6.35mm. The temperature of this test is -6°C, -12°C and -18°C. The test process is shown in Fig 6. During the test, a vertical load of 0.98N±0.05N was applied to the middle point of the beam for 240s, and the load and deformation were recorded for each 0.5s. Two indexes of creep stiffness S and creep rate m during the 60s were used to evaluate the low temperature performance of the asphalt binder. Formulas (10) and (11) were used to calculate creep stiffness S and creep rate m. It is generally believed that the smaller the S value, the softer the asphalt and the less likely it is to crack under low temperature conditions. The larger the m value, the better the stress relaxation ability of asphalt and the better the crack resistance of asphalt. The SHRP classification standard requires S to be no higher than 300MPa and m to be no less than 0.3 MPa.


$$S(t)=\frac{Pl^{3}}{4bh^{3}\delta (t)}$$ (10)

$$m(t)=|\frac{d\mathrm{lg}S(t)}{d\mathrm{lg}t}|$$ (11)

Where: S(t) is the creep stiffness at time t, MPa; P is the fixed load, N; l is trabecular support distance, 101.6mm; b is the beam width, 12.7mm; h is the beam thickness, 6.35mm; δ(t) is the beam deformation at time t, mm; m(t) is the creep rate at time t.

## 4 Results and discussion

### 4.1 Three indexes test

**Fig 7:**
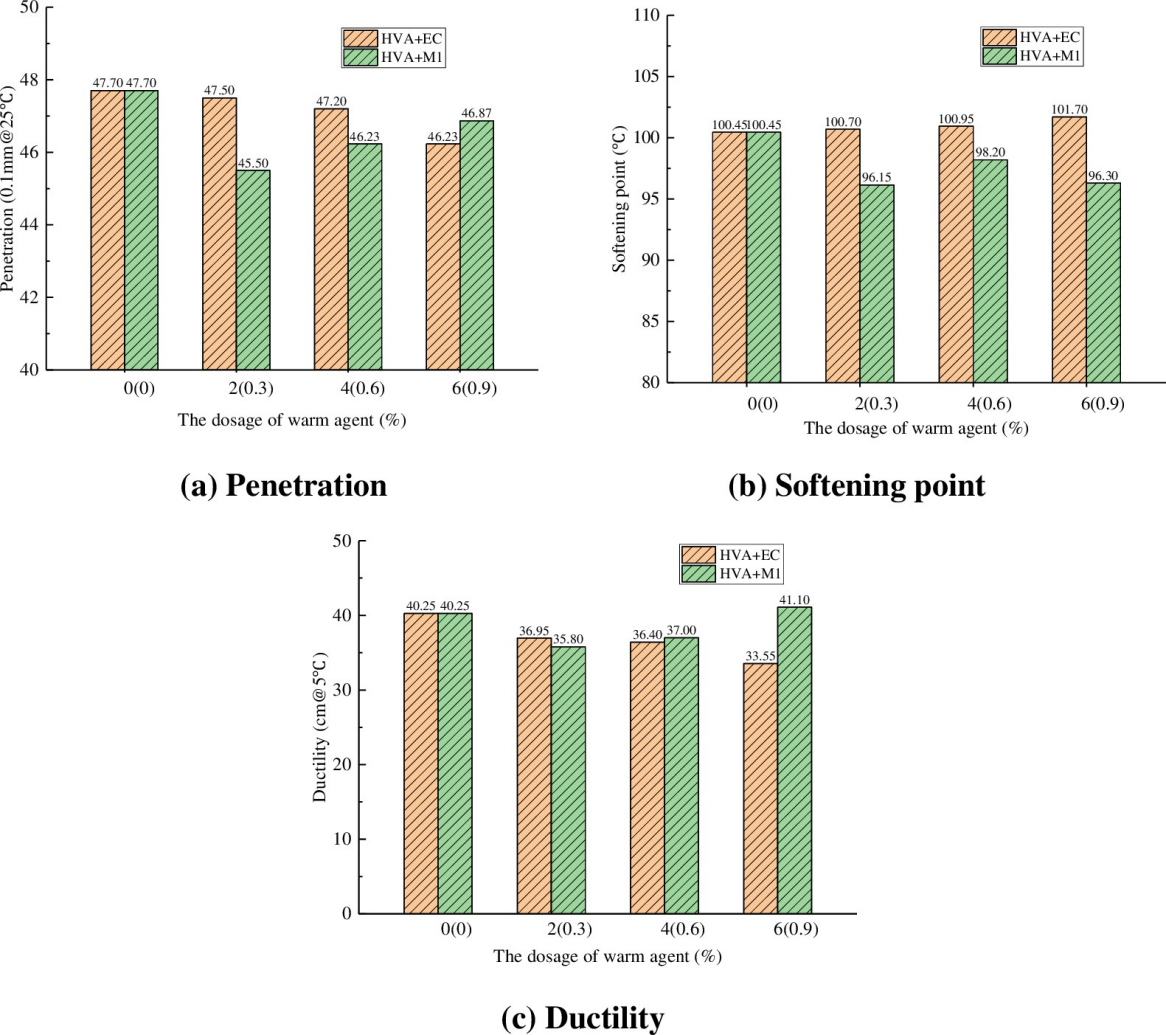
Three indexes of warm mixing HVA.

The test results of softening point, penetration and ductility are shown in Fig 7.

From Fig 7(A), it can be seen that the penetration of HVA decreases with the addition of EC, and the more the content, the smaller the penetration, indicating that the consistency of asphalt increases and the deformation resistance enhanced. The addition of M1 will also reduce the penetration of HVA, but it will gradually increase with the increase of dosage. From Fig 7(B), it can be seen that the softening point of HVA increases with the addition of EC, and also increases with the increase of dosage, indicating that EC can improve the high temperature stability of HVA. The addition of M1 significantly reduces the softening point of HVA, indicating that M1 will reduce the hightemperature stability of HVA. From Fig 7(C), it can be seen that the ductility of HVA decreases with the addition of EC, and decreases with the increase of dosage, indicating that EC will reduce the low temperature crack resistance performance of HVA. When the dosage of EC is 6%, the ductility decreases to 33.55cm, with a decrease of 16.6%. The addition of M1 will also reduce the ductility of HVA, but it will gradually increase with the increase of dosage, indicating that M1 can improve the low temperature performance of HVA.

### 4.2 Brinell rotary viscosity test

**Fig 8:**
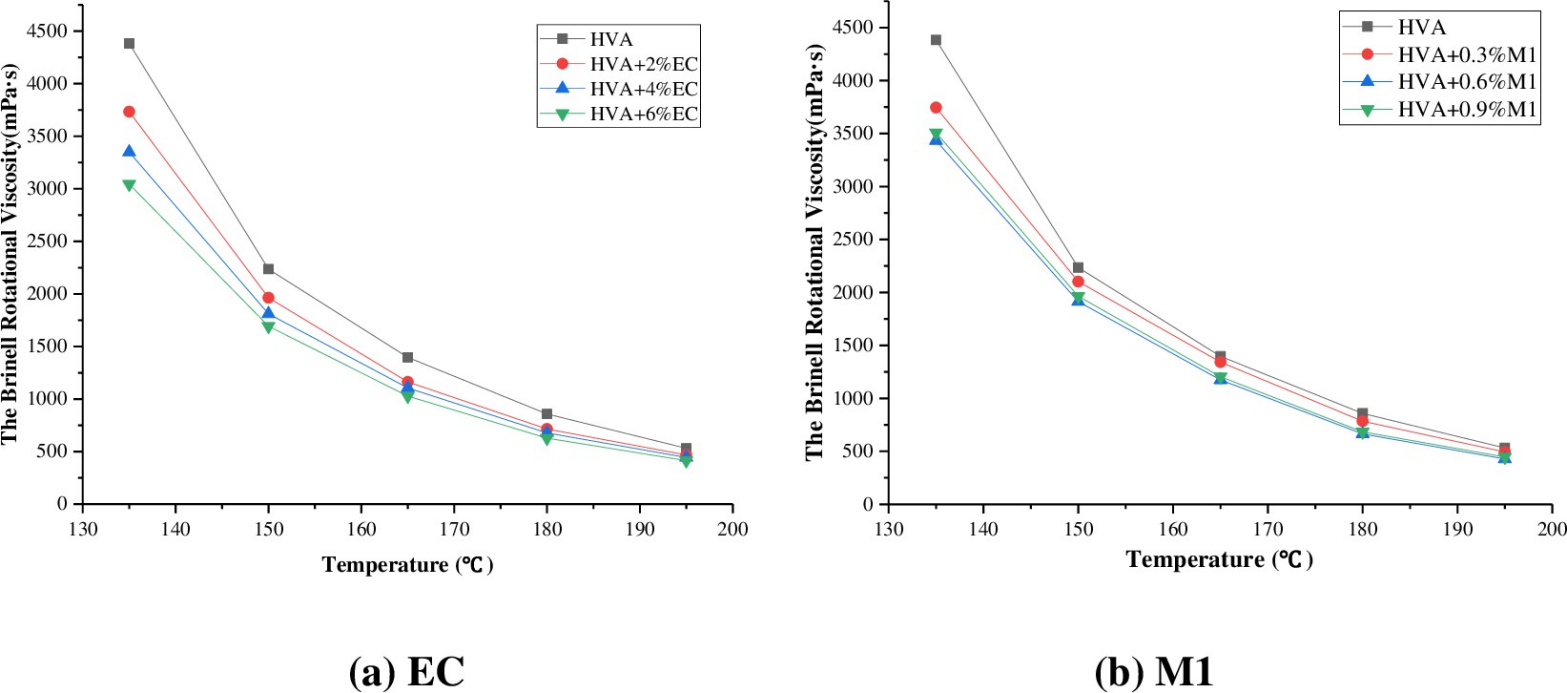
Viscosity-temperature curve of warm mix HVA.

The Brinell rotary viscosity test results of warm mixed HVA are shown in Table 3 and Fig 8.

**Table 3:** Test results of Brinell rotational viscosity (mPa·s).

Temperature	HVA	HVA+2%EC	HVA+4%EC	HVA+6%EC	HVA+0.3%M1	HVA+0.6%M1	HVA+0.9%M1
135°C	4383	3734	3349	3045	3746	3433	3508
150°C	2235	1964	1810	1693	2103	1914	1964
165°C	1396	1162	1105	1028	1343	1173	1203
180°C	858	715	678	628	784	665	684
195°C	531	468	444	414	494	428	446

It can be seen from Fig 8 that the Brinell viscosity of asphalt decreases with the increase of temperature, and the viscosity decreasing rate is initially fast and then tends to slow down. When EC and M1 are added into asphalt, the viscosity is smaller than that of HVA without warm mixing agent at the same temperature, and the more warm mix agents are added, the lower the viscosity of the HVA. This is mainly because the warm mixing agent plays a lubricating role, thereby reducing the viscosity of the asphalt. At 135°C, the addition of 6% EC can reduce the viscosity of HVA by 30%, and the addition of 0.9%M1 can reduce the viscosity of HVA by 20%, indicating that both warm mix agents have viscosity reduction effect and can reduce the construction temperature.

Formula (12) can be obtained by fitting the viscosity temperature curve of asphalt with semi-logarithmic coordinates.


lnη=A‐B·T (12)

**Fig 9:**
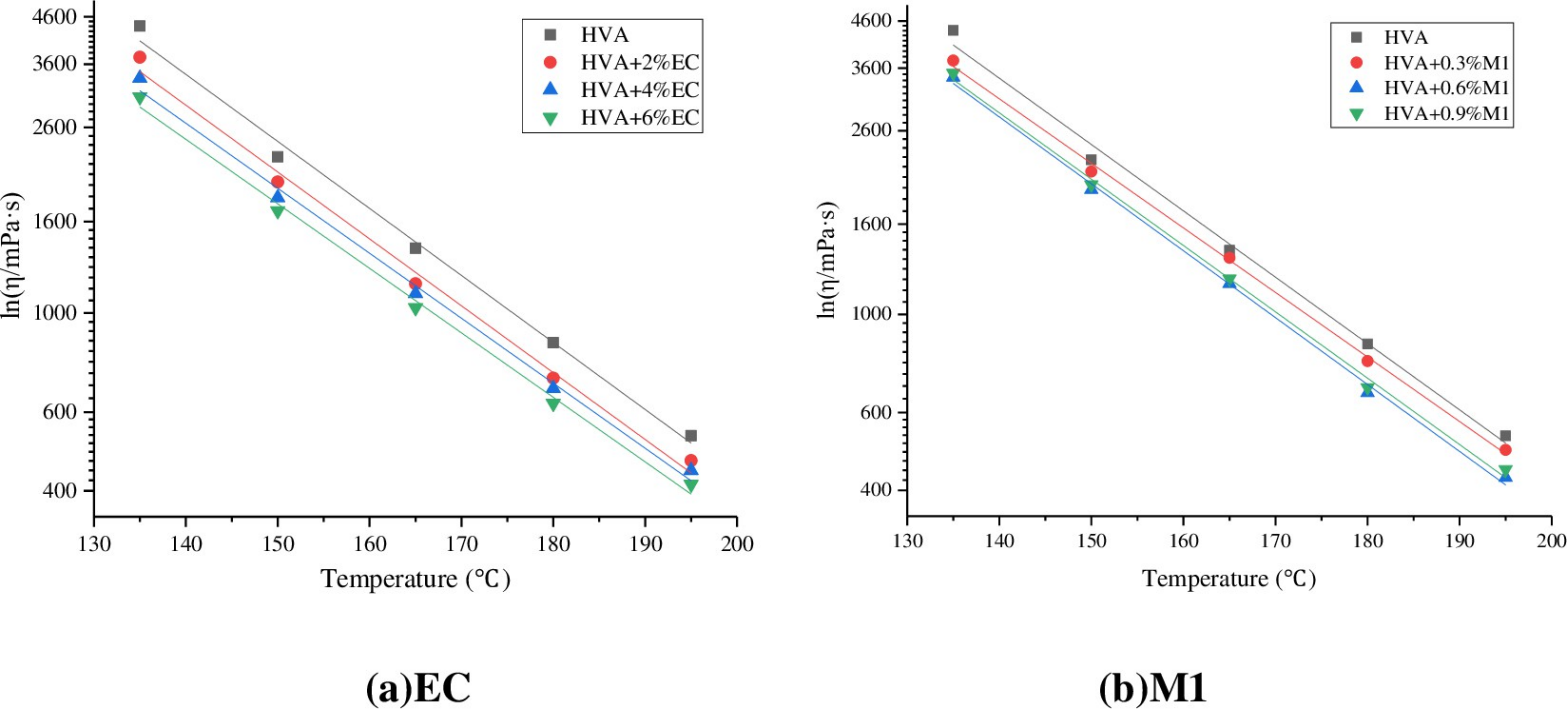
Semi-logarithmic fitting of viscosity of warm mix HVA.

Where: η represents viscosity, T represents temperature, and A and B are fitting parameters. The value of B reflects the sensitivity of asphalt viscosity to temperature change, and the larger the value of B, the more sensitive the viscosity is to temperature change. The fitting curves and parameters are shown in Fig 9 and Table 4.

**Table 4:** Regression parameters of viscosity-temperature curve.

Asphalt type	Fitting equation			
	lnη = A-B·T	A	B	R2
HVA	lnη = 12.970–0.035T	12.970	0.035	0.9946
HVA+2%EC	lnη = 12.780–0.034T	12.780	0.034	0.9938
HVA+4%EC	lnη = 12.572–0.033T	12.572	0.033	0.9954
HVA+6%EC	lnη = 12.454–0.033T	12.454	0.033	0.9964
HVA+0.3%M1	lnη = 12.732–0.033T	12.732	0.033	0.9985
HVA+0.6%M1	lnη = 12.810–0.034T	12.810	0.034	0.9981
HVA+0.9%M1	lnη = 12.790–0.034T	12.790	0.034	0.9977

It can be seen from Table 4 that the correlation coefficients of the fitting results are all around 0.99, indicating that the semilog function can well show the viscosivy-temperature variation characteristics of asphalt at different temperatures. The slope difference of the viscosity-temperature fitting line is small, and the fitting lines are almost parallel, indicating that the warm mixing agent has little effect on the viscosity-temperature sensitivity of the HVA.

### 4.3 Dynamic shear rheological test

#### 4.3.1 Temperature scanning

**Fig 10:**
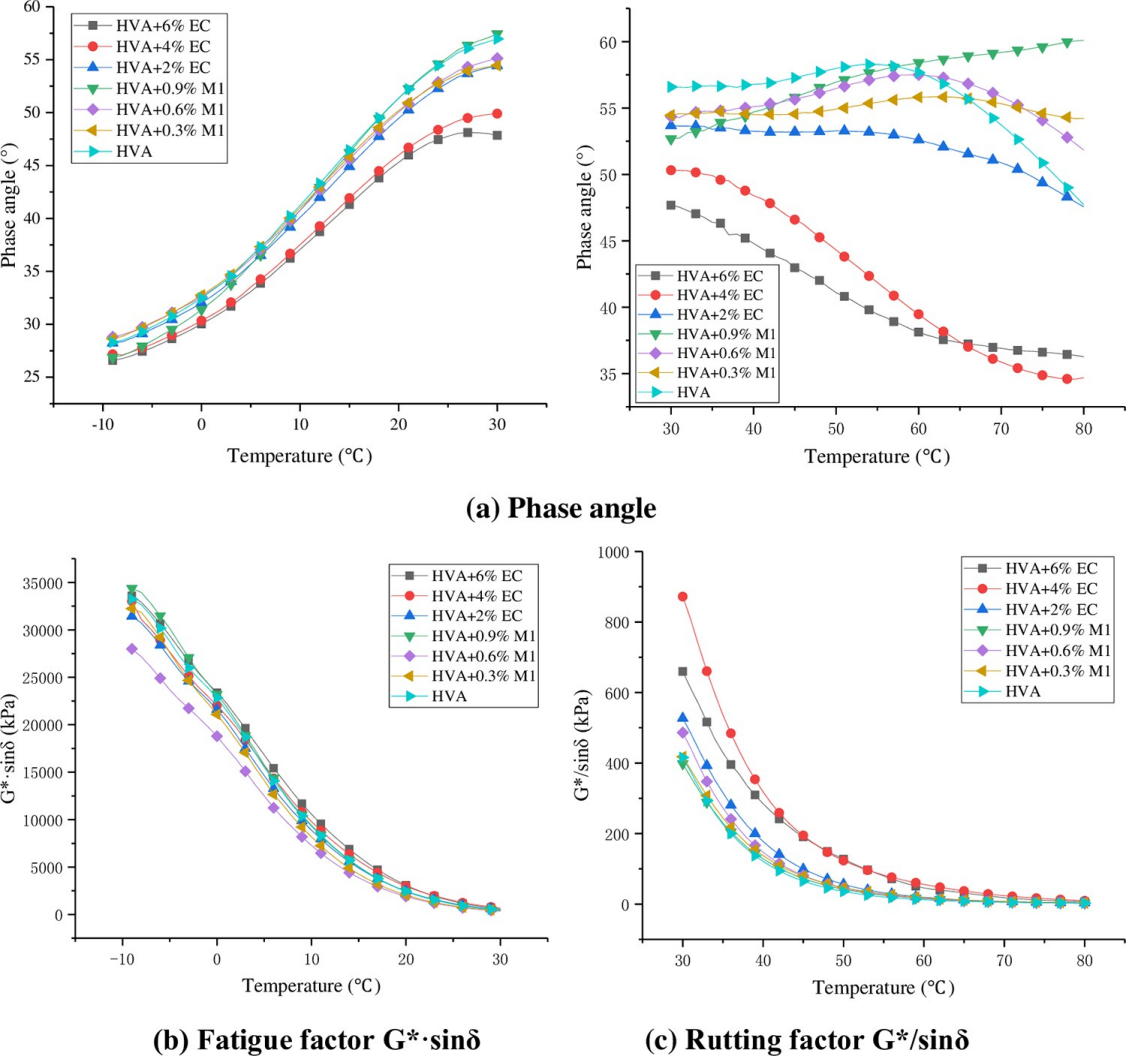
Test results of temperature scanning.

Through temperature scanning test, the results of phase Angle, fatigue factor G*·sinδ and rutting factor G*/sinδ of the warm mixed HVA were obtained, as shown in Fig 10.

As can be seen from Fig 10(A), when the temperature ranges from -10°C to 30°C, the phase Angle of asphalt will increase with the increase of temperature. This is because the asphalt will change from hard to soft with the increase of temperature, and the viscous part of asphalt will increase while the elastic part will decrease. The addition of M1 has little effect on the phase Angle, but the addition of EC reduces the phase Angle. This is mainly because EC is a solid state at room temperature and M1 is a liquid state. When the temperature is in the range of 30°C~80°C, the phase Angle of EC warm mixed HVA decreases significantly, while the phase Angle of M1 warm mixed HVA does not change much. This is mainly because EC is a straight-chain aliphatic hydrocarbon, which is solid at low temperature, forming a stable grid structure in the asphalt, increasing the elasticity of the asphalt, resulting in a decrease in phase Angle.

As can be seen from Fig 10(B), the fatigue factor G*•sin*δ* decreases gradually with the increase of temperature, which indicates that the fatigue resistance of asphalt at high temperature is better than that at low temperature. The fatigue factor of HVA decreased after the addition of M1, and the fatigue factor was the smallest when the dosage was 0.6%. The fatigue factor increased after the addition of EC, and the fatigue factor reached the maximum when the dosage was 6%. The results show that M1 can increase the fatigue resistance of asphalt, while EC can reduce the fatigue resistance of asphalt.

As can be seen from Fig 10(C), the rutting factor G*/sinδ decreases with the increase of temperature, indicating that the rutting resistance of asphalt decreases at high temperature. The rutting factor of HVA mixed with M1 has little change, while the rutting factor of HVA mixed with EC will increase significantly. When the content of EC is 4%, the high temperature rutting resistance of HVA is the strongest. This indicates that EC can significantly improve the rutting resistance of HVA.

#### 4.3.2 Frequency scanning

**Fig 11:**
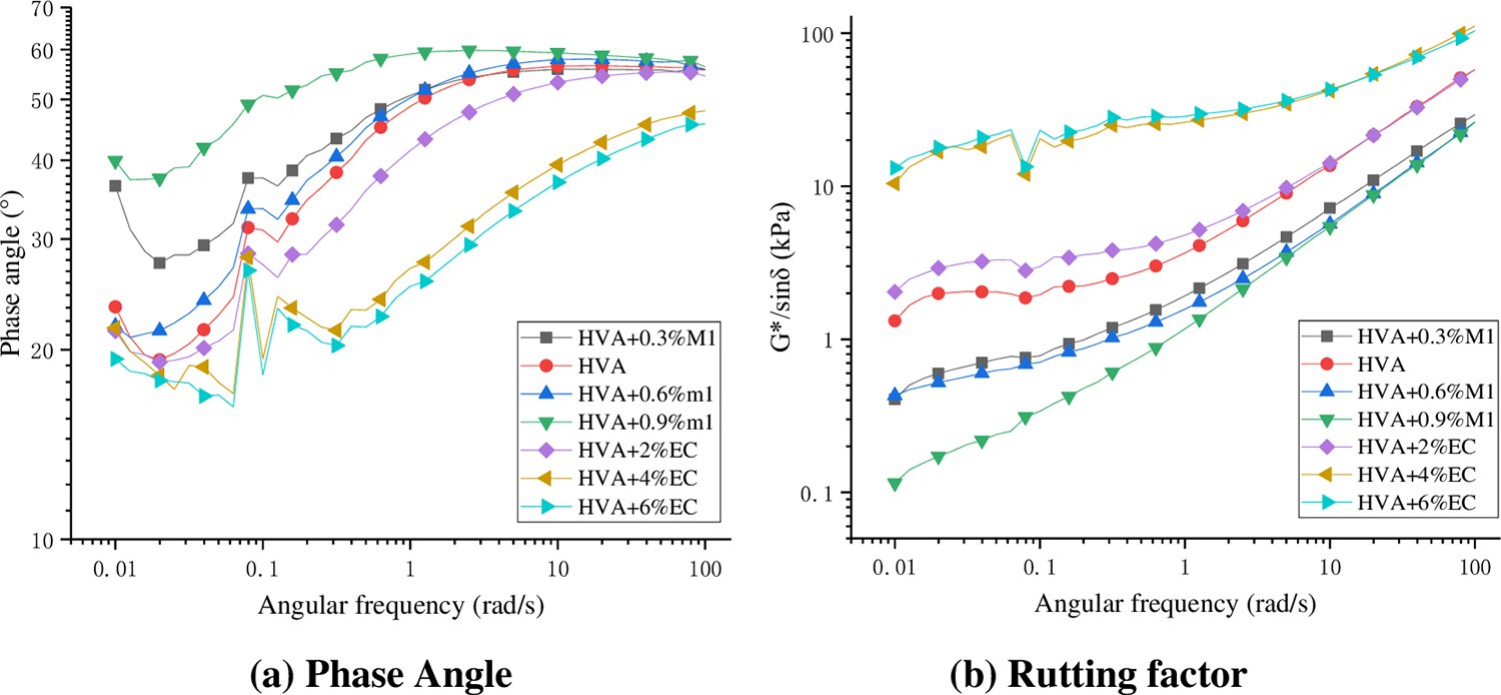
Test results of frequency scanning.

Through the frequency scanning test, the variation rules of phase Angle and rutting factor of the warm mixed HVA at different frequencies were obtained, as shown in the double logarithmic coordinate Fig 11.

As can be seen from Fig 11, the phase angle and rutting factor of HVA generally increase with the increase of shear frequency, indicating that the elastic ratio of HVA decreases and the viscosity ratio increases under high-frequency loading, and the anti rutting deformation ability of HVA is enhanced. That is to say, HVA is more prone to deformation under low-frequency loading, which is also the reason why asphalt pavement is used to produce rutting diseases in sections with slow driving speeds such as long longitudinal slopes and traffic congestion. The addition of M1 increases the phase angle of HVA and reduces the rutting factor, especially in the low-frequency state where the amplitude of phase angle increase and rutting factor decrease is more obvious. The addition of EC makes the asphalt exhibit the opposite phenomenon to M1. In each frequency range, the phase Angle of HVA will become smaller and the rutting factor will increase significantly, which greatly improves the high temperature performance of HVA.

#### 4.3.3 Linear amplitude scanning

**Fig 12:**
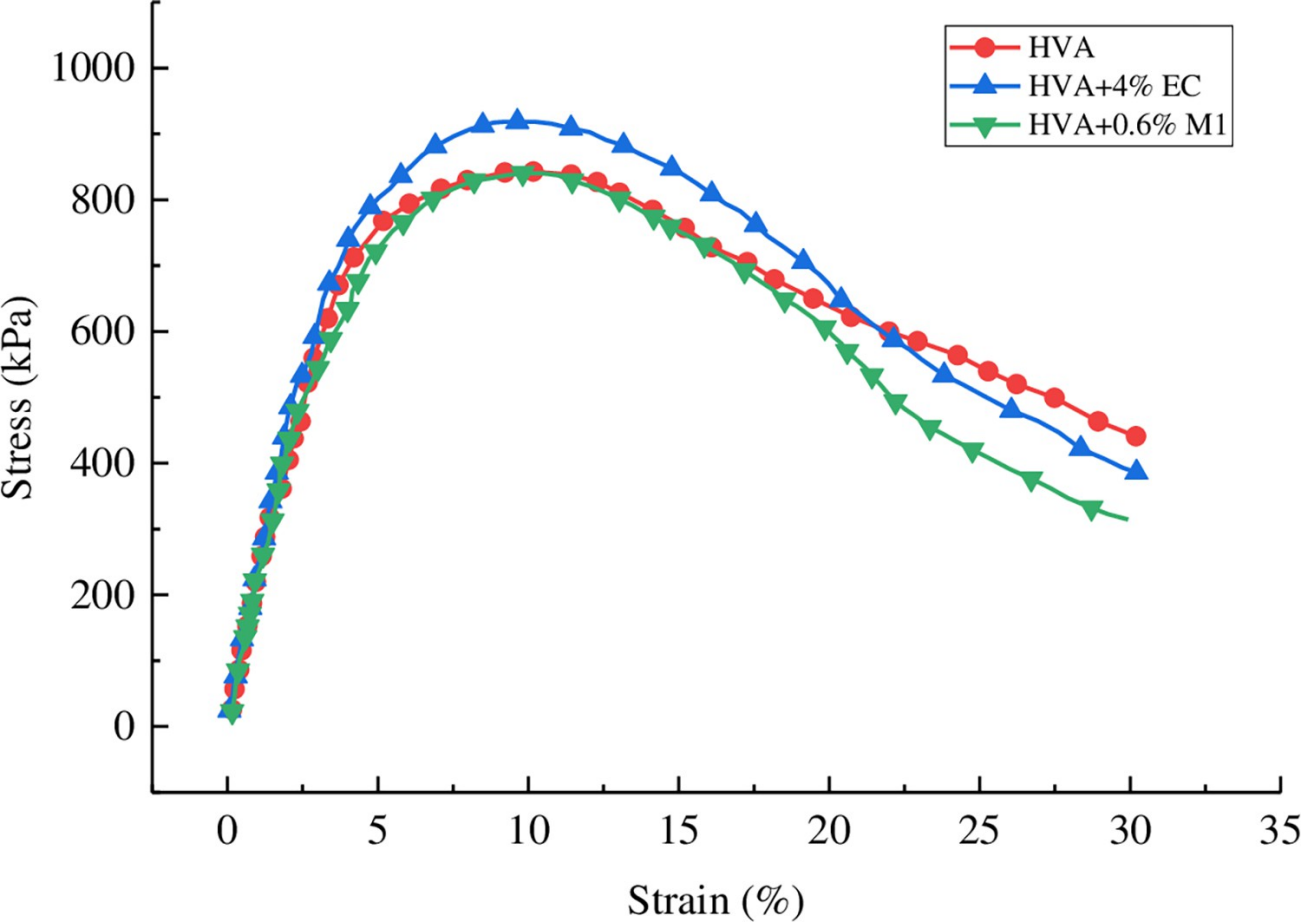
Stress-strain curve of HVA with linear amplitude scanning test.

The stress-strain curve of asphalt obtained through linear amplitude scanning test is shown in Fig 12.

As can be seen from Fig 12, the shear stress of asphalt increases first and then decreases with the increase of strain, with an obvious peak value. The peak stresses of HVA, 0.6% M1 warm mix HVA, and 4% EC warm mix HVA are 843 kPa, 840 kPa, and 919 kPa, respectively, and the corresponding strain values of peak stress are 10.2%, 10.4% and 9.2%. The results show that M1 can improve the ductility and toughness of asphalt, while EC has the opposite effect.

**Fig 13:**
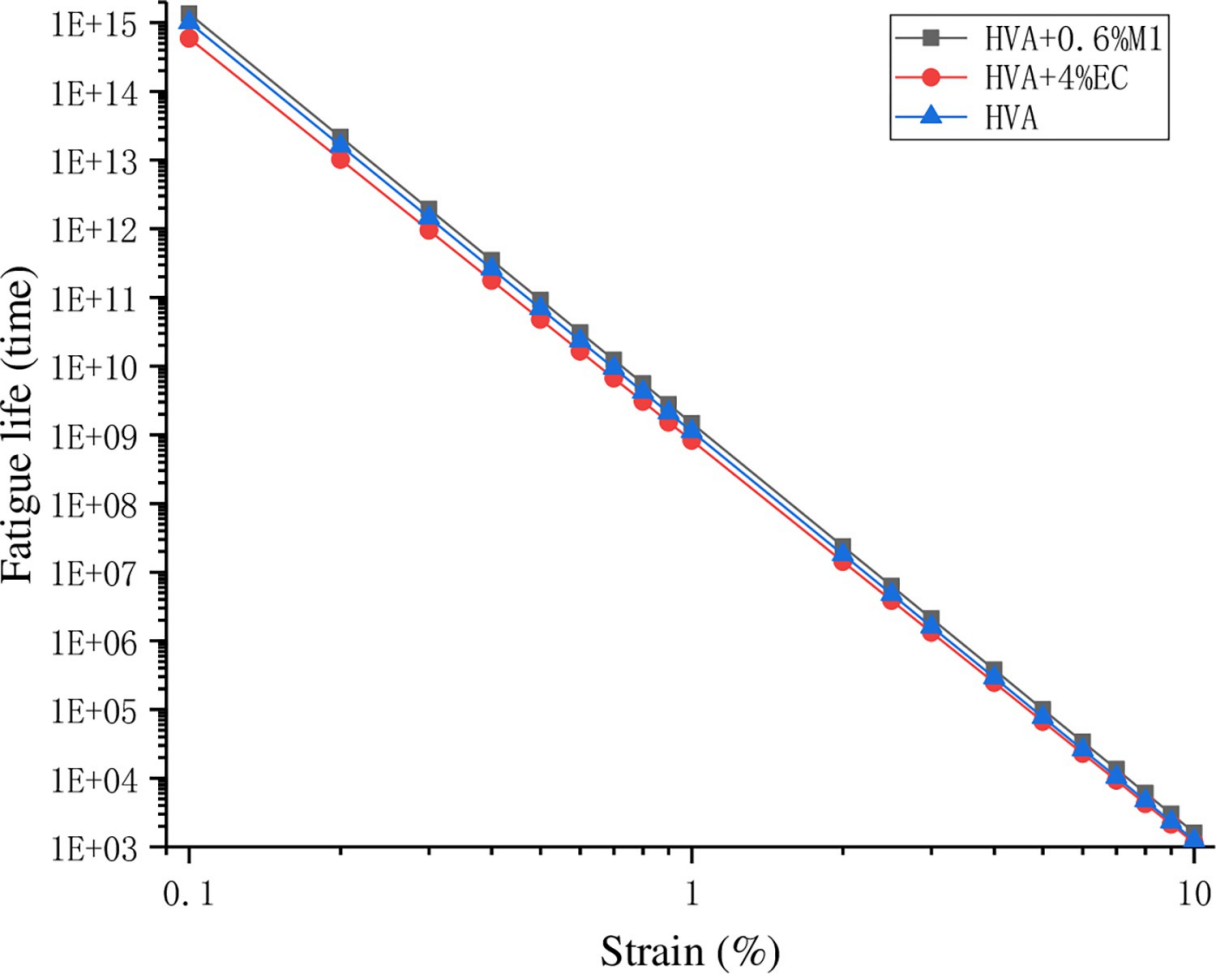
The logarithmic coordinate diagram between fatigue life and strain.

According to the fatigue life model, various parameter indicators are calculated and shown in Table 5. The fatigue life of asphalt under different strains is shown in Table 6. The logarithmic coordinate relationship between fatigue life and strain is shown in Fig 13.

**Table 5:** Parameters of asphalt LAS test model.

Asphalt	*α*	*C*_1_	*C*_2_	*D*_*f*_	*k*	*B*	*A*_35_
HVA	2.977	0.173	0.440	12752.47	2.667	5.954	1116596979
HVA+0.6% M1	2.981	0.169	0.438	13139.37	2.675	5.962	1474346356
HVA+4% EC	2.928	0.196	0.435	14248.50	2.654	5.856	823396996

**Table 6:** Fatigue life of asphalt under different strains.

Strain level (%)	Fatigue life (times)		
	HVA	HVA+0.6% M1	HVA+4%EC
0.1	1.004E+15	1.351E+15	5.9103E+14
0.2	1.62E+13	2.167E+13	1.0204E+13
0.3	1.449E+12	1.932E+12	9.497E+11
0.4	2.614E+11	3.476E+11	1.7618E+11
0.5	6.922E+10	9.191E+10	4.7691E+10
0.6	2.338E+10	3.099E+10	1.6397E+10
0.7	9.336E+09	1.236E+10	6648369614
0.8	4.216E+09	5.577E+09	3041685549
0.9	2.091E+09	2.763E+09	1526037279
1	1.117E+09	1.474E+09	823396996
2	18012079	23651500	14216007
2.5	4770475	6252896	3848332
3	1611078	2108641	1323083
4	290557	379418	245440
5	76954	100309	66442
6	25989	33827	22843
7	10380	13494	9262
8	4687	6087	4238
9	2325	3016	2126
10	1241	1609	1147

It can be seen from Table 6 and Fig 13 that the fatigue life of asphalt decreases sharply with the increase of strain. M1 will increase the fatigue life of HVA, while EC will reduce the fatigue life. After addition of 0.6% M1 warm mix agent, the fatigue life of HVA will increase by about 30% on average, while after addition of 4% EC warm mix agent, the fatigue life will decrease by about 9% on average. This is mainly because M1 is a surfactant and liquid, which can change the surface tension between asphaltene molecules, which is beneficial for fatigue resistance. EC is a waxy warm mix agent, showing a crystal state at low temperature, which can improve the high temperature performance of asphalt but reduce the low temperature performance and fatigue resistance.

According to the road strength, Hussain Bahia [27] suggested the grade of asphalt fatigue life as shown in Table 7, in which the pavement with high strength adopts 2.5% strain, while the pavement with low strength adopts 5% strain. It can be seen that the fatigue life grades of the three asphalt can meet the V and E grades, and the fatigue resistance of the asphalt is ranked as HVA+0.6% M1>HVA>HVA+4% EC.

**Table 7:** Fatigue life grade of LAS test.

Load level	S	H	V、E
*N*_*f2*.*5%*_ and *N*_*f5%*_	>15000	>19000	>31000

### 4.4 Bending beam rheological test

**Fig 14:**
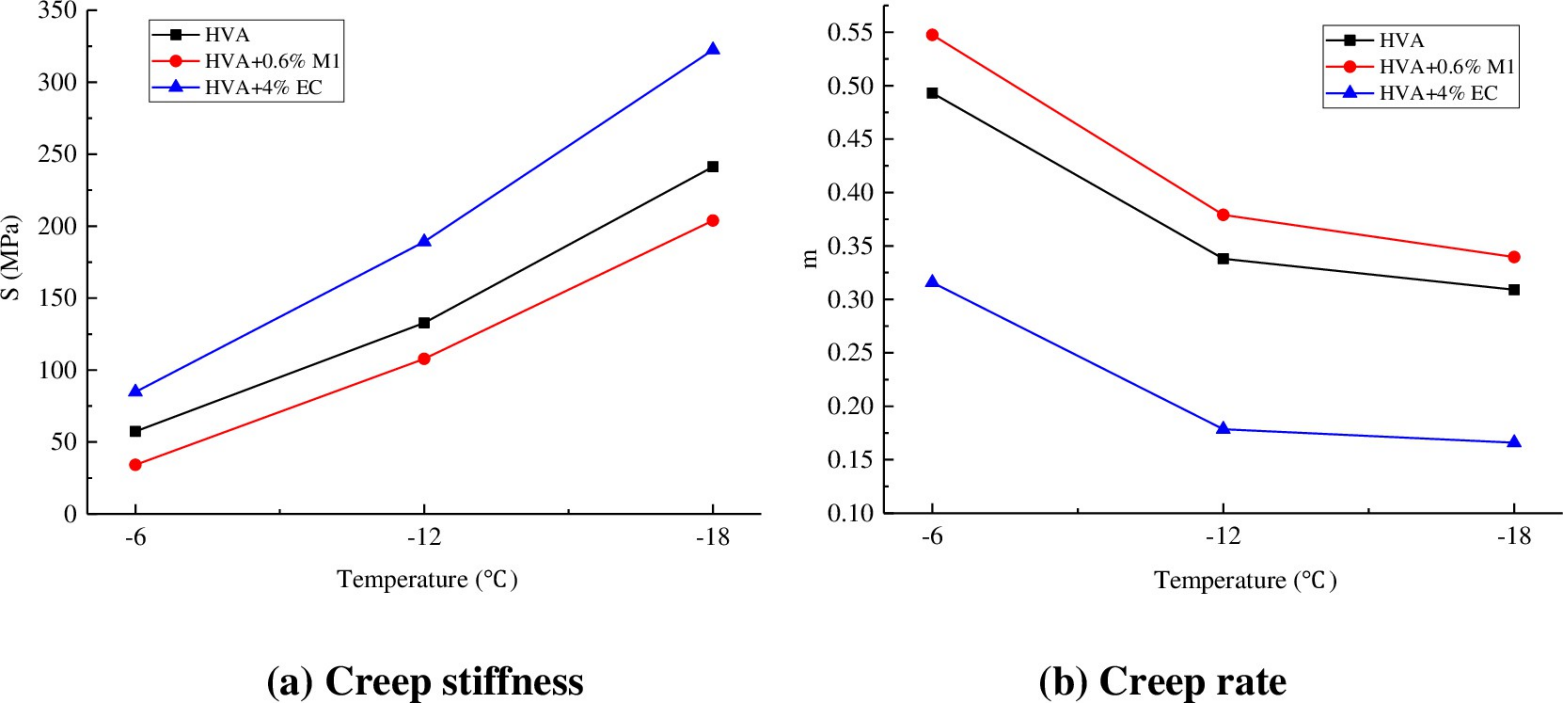
Results of creep test.

The creep test results of the low-temperature bending beam are shown in Fig 14.

It can be seen from Fig 14 that the creep stiffness S of asphalt increases with the decrease of temperature, and the creep rate m decreases with the decrease of temperature, mainly because the asphalt gradually hardens with the decrease of temperature. The addition of M1 reduced the creep stiffness S of HVA and increased the creep rate m, while EC showed the opposite effect, indicating that M1 improved the low temperature performance of HVA while EC weakened the low temperature performance of HVA. According to the requirements of S not higher than 300MPa and m not lower than 0.3 in the SHRP classification standard, only HVA and M1 warm mixed HVA can meet the requirements at the three test temperatures. The low temperature performance of asphalt binder is ranked as follows: HVA+0.6% M1>HVA>HVA+4% EC.

## 5 Conclusions

In this paper, the effects of EC and M1 warm mixing agent on the rheological properties of HVA at high, medium and low temperatures were studied by using Brinell rotary viscosity test, dynamic shear rheological test and low temperature bending beam creep test. The main conclusions are as follows:

1. Both EC and M1 warm mixing agents have good viscosity reduction effects on HVA, and the recommended optimal dosage of warm mixing agents is 4% EC and 0.6% M1.
2. M1 can effectively improve the fatigue performance of HVA, and the fatigue life is increased by about 30% when the dosage is 0.6%. EC can improve the rutting factor of HVA, but it will reduce the fatigue resistance, and the fatigue life will be reduced by about 9% when the dosage is 4%.
3. The addition of M1 warm mixing agent can reduce the creep stiffness S and increase the creep rate m, and improve the low temperature performance of HVA, while EC warm mixing agent shows the opposite effect, weakening the low temperature performance of HVA.
